# Gene expression profile of epithelial cells and mesenchymal cells derived from limbal explant culture

**Published:** 2010-07-06

**Authors:** Naresh Polisetti, Prasoon Agarwal, Imran Khan, Paturu Kondaiah, Virender S. Sangwan, Geeta K. Vemuganti

**Affiliations:** 1 Sudhakar and Sreekanth Ravi Stem Cell Biology Laboratory, C-TRACER, Hyderabad Eye Research Foundation, L.V. Prasad Eye Institute, Hyderabad, India; 2 Molecular Reproduction, Development and Genetics, Indian Institute of Science, Banglore, India; 3 Cornea and Anterior Segment Service, L.V. Prasad Eye Institute, Hyderabad, India

## Abstract

**Purpose:**

Limbal stem cell deficiency is a challenging clinical problem and the current treatment involves replenishing the depleted limbal stem cell (LSC) pool by either limbal tissue transplantation or use of cultivated limbal epithelial cells (LEC). Our experience of cultivating the LEC on denuded human amniotic membrane using a feeder cell free method, led to identification of mesenchymal cells of limbus (MC-L), which showed phenotypic resemblance to bone marrow derived mesenchymal stem cells (MSC-BM). To understand the transcriptional profile of these cells, microarray experiments were carried out.

**Methods:**

RNA was isolated from cultured LEC, MC-L and MSC-BM and microarray experiments were carried out by using Agilent chip (4×44 k). The microarray data was validated by using Realtime and semiquntitative reverse transcription polymerase chain reaction.

**Results:**

The microarray analysis revealed specific gene signature of LEC and MC-L, and also their complementary role related to cytokine and growth factor profile, thus supporting the nurturing roles of the MC-L. We have also observed similar and differential gene expression between MC-L and MSC-BM.

**Conclusions:**

This study represents the first extensive gene expression analysis of limbal explant culture derived epithelial and mesenchymal cells and as such reveals new insight into the biology, ontogeny, and in vivo function of these cells.

## Introduction

One of the most important advances made in translational research is in the field of ocular surface reconstruction using cell therapy [1-3]. This technology owes its success not only to the surgical advances but also to the increasing amount of knowledge pertaining to the location, characteristics and functioning of Limbal stem cells (LSC) [4-6]. In the normal uninjured state, LSC are mitotically quiescent and maintained in a specialized limbal stromal microenvironment or “niche.” However, upon corneal epithelial wounding, stem cells located in the limbus proliferate to generate more stem cells and transient amplifying cells to replace the damaged epithelium. It is generally agreed that the LSC are characterized by special location in the limbus, clonality, cytokeratin profile, transformation-related protein 63 (p63) delta isomers, and ATP-binding cassette sub-family G member 2 (ABCG2) expression [7-9]. It is well established that the niche plays an important role in the maintenance of stem cell properties in several tissues and this is expected to be true in the case of the LSC niche as well [10-13]. Some of the assumed factors for niche regulation include proximity to vasculature [14]; the basement membrane composition with respect to specific isoforms of collagen IV, laminin and fibronectin [15]; and the presence of limbal fibroblasts in the underlying stroma, which produce various cytokines [16].

We had earlier reported the presence of spindle shaped cells in extended limbal explant cultures, which bear a striking resemblance to the mesenhcymal stem cells derived from bone marrow (MSC-BM), which we had referred to as mesenhcymal like cells from limbus (MC-L) [17]. Interestingly, limbal fibroblast-like cells have also been reported to have stem cell like properties [18] and their conditioned media has been reported to foster conversion of human embryonic stem cells into corneal epithelial-like cells [19].

Several groups have reported the gene expression profile of limbal and corneal epithelial cells that has significantly contributed to the understanding of several cellular pathways and intrinsic factors that underpin the phenotypic difference between the two cell types [20-22]. These studies and the study by Zhou et al. [23], have used the native corneal and limbal tissue to derive the gene expression profile. However the gene expression profile of the cultured human limbal epithelial and stromal cells cultured cells obtained from the native limbal tissue that is used for clinical transplantation to regenerate the ocular surface has not been addressed until now. In the present study, we evaluated the transcriptome of the limbal explant culture derived epithelial and mesenchymal like cells by microarray and identified expression of unique genes and biologic pathways that characterize both these cell types. To evaluate our hypothesis that the MC-L possibly act as one of the “niche” derived intrinsic feeder cells in the feeder cell free method of limbal explants culture, we compared the profile of these cells to that of the MSC-BM, which form the supporting niche for the hematopoietic system.

## Methods

All the procedures, recruitment of patients and the protocol were approved by the Institutional Review Board (L.V. Prasad Eye Institute IRB, Hyderabad, India) and the research followed the tenets of the declaration of Helsinki.

### Establishment of cell cultures

In an ongoing clinical trial, which was approved by the IRB, limbal epithelial cultures were established from limbal biopsies as described in our previous publications [2,3,17]. Briefly, less than 1×2 mm^2^ piece of limbal biopsy was obtained from the limbal region which included the epithelium as well as 0.5 mm of stromal tissue. Limbal epithelial cultures were established on de-epitheliazed human amniotic membrane (dhAM) with the basement membrane side up, in a feeder cell-free culture system. Cultures were established in duplicates for each clinical sample of which one culture was used for transplantation (after 10–14 days in culture) while the other culture used for experiments. A total of 25 samples were used in this study of which, 3 samples were used for RNA isolation and the remaining were cultured further. The limbal explants cultures in the duplicate plates were incubated for 2–3 weeks more to propogate the adherent spindle cells in the bottom of the Petri dish, beyond the area of amniotic membrane, which contained epithelial cells (n=22). These plates adherent cells (epithelial and spindle cells) were then trypsinized and plated on a T25 flask (Thermofisher Scientific, Roskilde, Denmark) in DMEM medium supplemented with 10% fetal bovine serum (FBS; Sigma-Aldrich Inc., St. Louis, MO). The non-adherent epithelial cells suspended in the medium were removed by changing the medium. These cultures were maintained at 37 °C and 5% CO_2_ in humidified incubator (BINDER GmbH, Tuttlingen, Germany). When the cells reached 80%–90% confluence, cultures were harvested with 0.25% trypsin (Sigma-Aldrich) in 1mM EDTA solution (Sigma-Aldrich). MSC-BM cells were obtained using a previously described protocol [17]. Briefly, human MSC-BM cultures were established from bone marrow aspirates of healthy donors after obtaining informed consent. The bone marrow mononuclear cells (BMMNCs) were separated using Ficoll-Hypaque gradient at 400× g for 30 min. The mononuclear cells were then plated at a density of 1×10^7^ cells in Dulbecco's Modified Eagle's Medium (DMEM; Sigma-Aldrich Chemie, Steinheim, Germany) supplemented with 10% FBS. When cultures reached confluence, cells were passaged using trypsin-EDTA in line 100.

### Characterization of cells

#### Immunocytochemical analysis

Immunocytochemical analysis was performed as previously described [17]. Briefly, cells were incubated with primary antibodies for vimentin, ATP-binding cassette subfamily G member 2 (ABCG2), Cytokeratin 14 (KRT14), Cytokeratin 3/12 (KRT3/12), E-cadherin (CDH1), cytokeratin 19 (KRT19), cluster of differentiation 45 (CD45), and nestin (Chemicon, Billerica, MA). Double immunostaining was performed for vimentin and paired box gene 6 (PAX-6). Following incubation with secondary antibodies (conjugated to Fluorscein Isothiocynate (FITC) or Tetramethyl Rhodamine Isothiocynate (TRITC), the nucleus was counterstained with propidium iodide (PI). The stained preparations were screened with a laser scanning confocal microscope (LSM 510; Carl Zeiss, Jena, Germany) using a fluorescent light source.

#### Flow cytometry analysis

MC-L were characterized for the expression of CD44-PE (Phycoerythrin), CD90-FITC, CD13-FITC, human leukocyte antigen (HLA-ABC-PE and HLA-DR-PE), CD10-PE, CD40-FITC, CD11b-FITC, CD40L-APC (Allophycocyanin), CXC chemokine receptor 4 (CXCR4) –APC (eBioscience^TM^, San Diego, CA) CD34-FITC, CD138-PerCP (Peridinin Chlorophyll Protein Complex; BD Pharmingen™, San Diego, CA) markers. Briefly, a single cell suspension of 0.5 to 1×10^6^ cells obtained in 100 µl of PBS (phosphate buffered saline) containing 0.1% sodium azide and 2% FBS. These were incubated with saturating concentrations of the respective primary antibodies or conjugated antibodies for 45 min. After three washes, the cells were centrifuged at 200× g for 5 min and resuspended in ice-cold PBS. Fluorescence was evaluated by BD-FACS Aria (BD Biosciences) and data were analyzed by using FACS Diva software. Corresponding isotype controls were included in each experiment and specific staining was measured from the cross point of the isotype with a specific antibody graph. A total of 20,000 events were acquired to determine the positivity of different cell surface markers used.

### Total RNA isolation

Total RNA was extracted from each sample (n=3 for LEC, MC-L, and MSC-BM) with Trizol (Invitrogen, Carlsbad, CA) and purified using RNAeasy Columns (Qiagen GmbH, Hilden, Germany,). RNA was quantitated by NanoDrop ND-1000 (Thermo Scientific, Wilmington DE) and the quantity and integrity was established by resolving on 0.8% formaldehyde agarose gels.

### Expression profiling of genes by microarray experiments

Microarray experiments were performed using Agilent 4×44k oligonucleotide arrays. These arrays cover the entire genome. For labeling reaction, 500 ng of RNA from LEC, MC-L, and MSC-BM was used (n=2 of each LEC, MC-L, and MSC-BM). Labeling was done using the Quick Amp labeling kit (Agilent technologies, Santa Clara, CA) as per the manufacturer’s protocol. Briefly, using T7 promoter element coupled oligodT primer, cDNA was generated and from cDNA, labeled cRNA was generated via an in vitro transcription reaction using T7 RNA polymerase and Cy3 (for LEC and MC-L) or Cy5 (MC-L and MSC-BM) CTP. Labeled cRNA (825 ng) of the respective sample was used for hybridization in the following combinations - LEC (Cy3) and MC-L (Cy5), LEC (Cy3) and MSC-BM (Cy5) and MC-L (Cy3) and MSC-BM (Cy5). Hybridization was performed for 17 h, rotating at a speed of 10 rpm at 65 °C in an hybridization oven (Agilent Technologies).

### Microarray image and data analysis

Microarray image analysis was done using Feature extraction version 9.5.3.1 (Agilent Technologies) and data analysis was done using Gene Spring version 10 (Agilent Technologies). The background corrected intensity values were used for analysis. Normalization was done using LOWESS algorithm. Similarly expressed genes were filtered on the basis of standard deviation between two biologic replicates with the cut off of less than one. Fold changes were calculated and genes with more than twofold difference were selected.

### Validation of microarray using aemi quantitative RT–PCR and Real-time-PCR

To confirm the gene expression profile determined by microarray, several selected genes were subjected to RT–PCR analysis, using total RNAs derived from the two independent samples of LEC, MC-L, and MSC-BM that were used for the microarray experiments, as well as an additional pair of LEC, MC-L, and MSC-BM samples. Ribosomal protein large 35 (RPL35) a, a ribosomal protein served as an internal control. A 2 µg quantity of RNA was reverse transcribed using a cDNA synthesis kit (Applied Biosystems, Foster City, CA) and 1/100th of the reaction was used per 20 µl PCR reaction. PCR reactions were performed with DyNAZYME master mix (Finnzymes Oy, Espoo, Finland). The PCR products were resolved on a 2% agarose gel containing ethidium bromide. Real-Time PCR quantitation was performed in an ABI prism 7900 HT sequence detection system and analyzed with SDS 2.1 software (Applied Biosystems). The reactions were identical to those described above, except that DyNAMO^TM^SYBERgreen 2× mix (Finnzymes Oy) was used in place of DyNAZYME MIX. The sequences of primers are shown in Table 1. Amplification of RPL35a was performed for each cDNA (in triplicate) for normalization of RNA content. Threshhold cycle number (C_t_) of amplification in each sample was determined by ABI Prism Sequence Detection System software (Applied Biosystems). Relative mRNA abundance was calculated as the average for C_t_ for amplification of a gene-specific cDNA minus the average C_t_ for RPL35a and fold change over control has been calculated as follows:

**Table 1:** Sequence of the primer used for RT–PCR amplification of selected genes to substantiate the results obtained by microarray.

**Sample number**	**Gene name**	**Forward primer (5′→3′)**	**Reverse primer (5′→3′)**	**Product length (bp)**
1	Vascular Endothelial Growth factor A (VEGF-A)	ATGCGGATCAAACCTCACC	ATCTGGTTCCCGAAACCCTG	
	183			358
	165			304
	148			269
	121			172
2	Fibroblast Growth Factor 2 (FGF2)	GGAGAAGAGCGACCCTCAC	GTGCCACATACCAACTGGTG	221
3	(Angiopoietin 1 (Ang-1)	CCCAGAAACTTCAACATCTGG	GGACTGTGTCCATCAGCTC	537
4	Transforming growth factor, beta 1 (TGFB1)	GACTACTACGCCAAGGAGGTC	TCAACCACTGCCGCACAACTC	332
5	Nestin (NES)	CACCTGTGCCAGCCTTTCTTAA	CCACCGGATTCTCCATCCTTA	361
6	Neuron-specific class III beta-tubulin (Tuj1)	TCAAGCGCATCTCCGAGCAG	ACCGTAAAACGTCAGGCCTGGAG	444
7	Collagen 1 alpha 1	TCCCCAGCCACAAAGAGTCTA	TTTCCACACGTCTCGGTCA	201
8	Vimentin`	CAGGAACAGCATGTCCAAATCG	TGTACCATTCTTCTGCCTCCTGC	127
9	S100A2	GATCCATGATGTGCAGTTCTCT	GTTCTGCTTCAGGGTCGGT	310
10	RPL35A	GAACCAAAGGGAGCACACAG	CAATGGCCTTAGCAGGAAGA	236
11	PAX-6	GAATCAGAGAAGACAGGCCA	GGTAGGTATCATAACTCCG	302
12	S100A4	GATGAGCAACTTGGACAGCAA	CTGGGCTGCTTATCTGGGAAG	123
13	E-cadherin	AAGGTGACAGAGCCTCTGGATAGA	TCTGATCGGTTACCGTGATCAA	124
14	CD24	AACTAATGCCACCACCAAGG	CCTGTTTTTCCTTGCCACAT6	188
15	PBX-1	ACCCTTCGCCATGTTATCAG	ATTGCTGGGAGATCAGTTGG	189
16	OTX-1	CTCCACCCAGCTGTTAGCAT	CGCATGAAGATGTCAGGGTA	221
17	FOXA1	AGGGCTGGATGGTTGTATTG	AGGCCTGAGTTCATGTTGCT	150
18	SHC3	GACATCTACAGCACGCCAGA	CAAGGGCTGGTTCTTGAGAG	186
19	FOXF1	TTGGCAATATTTGCCGTGTA	CTGCACTCTAGCAGCCAAAA	209
20	CDH6	TCGAGAAAACAGGGAGCAGT	CGGTGGAGAAGATTCAGGAG	175
21	CDH11	GTGCCTGAGAGGTCCAATGT	GGGTAGGGCTGTTCTGATGA	165
22	Collagen VI alpha 1	ACAGTGACGAGGTGGAGATCA	GATAGCGCAGTCGGTGTAGG	122
23	Collagen IV alpha 2	TTGGCGGGTGTGAAGAAGTTT	CCTTGTCTCCTTTACGTCCCTG	178
24	IL-1B	GGGCCTCAAGGAAAAGAATC	TTCTGCTTGAGAGGTGCTGA	205
25	Fibronectin 1	GCAGTAACCACTATTCCTGCAC	TCCTGATACAACCACGGATGAG	192
26	T-cell differentiation protein 2 (MAL2)	TTGCCTCCTCCAATGTTCCTC	CAGTTAGCATCAATTTGAGCCAC	133
27	CTGF	CAGCATGGACGTTCGTCT	CCAACCACGGTTTGGTCCTT	117
28	SPARC (osteonectin)	CGAGACCTGTGACCTGGACAATG	TCCGGTACTGTGGAAGGAGTGG	127
29	Sflt-F	TGAGCACTGCAACAAAAAGG	TCCTCCGAGCCTGAAAGTTA	172
30	FLT-F	GGCTCTGTGGAAAGTTCAGC	GCTCACACACTGCTCATCCAAA	223
31	FGFR1, transcript variant 2 mRNA	TCCAGTGCATCCATGAACTCT	CTGTTGCGTCCGACTTCAA	268
32	Brain derived nerve growth factor	GATGCTCAGTAGTCAAGTGCC	GCCGTTACCCACTCACTAATAC	168
33	Chemokine (C-C motif) ligand 2	CAGCCAGATGCAATCAATGCC	TGGAATCCTGAACCCACTTCT	190
34	Chitanase 3 like 1	GAAGAGGCCCTGTCTAGGTA	AGATGATGTGGGTACAGAGG	250
35	Matrix Metallo proteinase 2 (MMP2)	CCGTCGCCCATCATCAAGTT	CTGTCTGGGGCAGTCCAAAG	169
36	Interleukin 1 aplha (IL-1A)	TGTGACTGCCCAAGATGAAG	CGCCTGGTTTTCCAGTATCT	238
37	Decorin	AGTTGGAACGACTTTATCTGTCC	GTGCCCAGTTCTATGACAATCA	160
38	Neurotrophin tyrosine kinase receptor 2	GATAAGCTGGACTCGGCACG	GGACGACATCCCTAGCAGCC	152
39	Connexin 43	TGTCCTTAAGTCCCTGCTAA	GTAGCTGAGGAATGATGAAAAAG	245

$$\Delta \text{ct}={\text{ct}}_{\text{gene}}-{\text{ ct}}_{\text{RPL}}$$

ΔΔct =Δct (one cell type) –Δct (another cell type)

$$\text{Fold Change }={\text{ 2}}^{-\Delta \Delta \text{ct}}$$

Three individual gene-specific values thus calculated were averaged to mean±standard deviation, and fold change was expressed as log 2 ratios.

## Results

### Establishment of cell cultures

**Figure 1:**
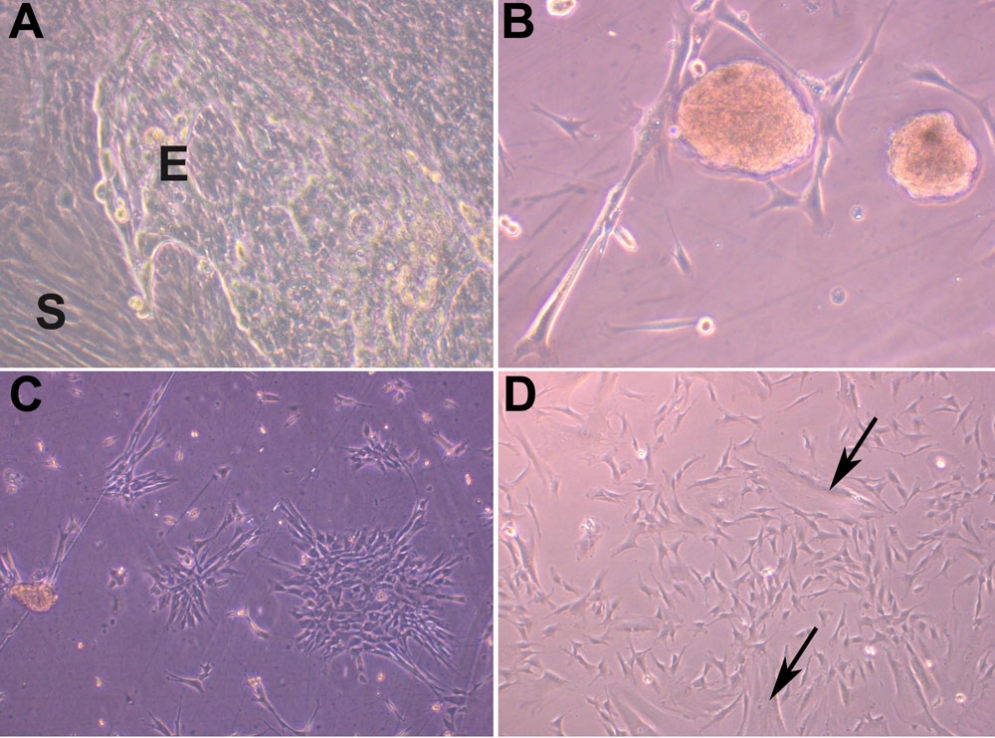
Morphological features of mesenchymal cells of limbus. Limbal explant cultures having epithelial (E) and mesenchymal cells (S; 200×; **A**). Cell sphere formation in the MC-L cultures giving impression of embryoid body formation (200×; **B**). Spindle shaped morphology of MC-L forming colonies (200×; **C**). Culture of MC-L showing both spindle shaped and broad flattened cells (arrows; 200×; **D**)

Spindle cell cultures were established from extended limbal explant cultures after 2 to 3 weeks of culture. Under a phase contrast microscope the cells appeared fibroblastic, elongated, and spindle shaped and few cells were large and flat with a single nucleus. These cells demonstrated the ability to form colonies with the occasional cell sphere formation giving the impression of embryoid bodies (Figure 1).

### Characteristics of limbal explant culture derived mesenchymal cells

**Figure 2:**
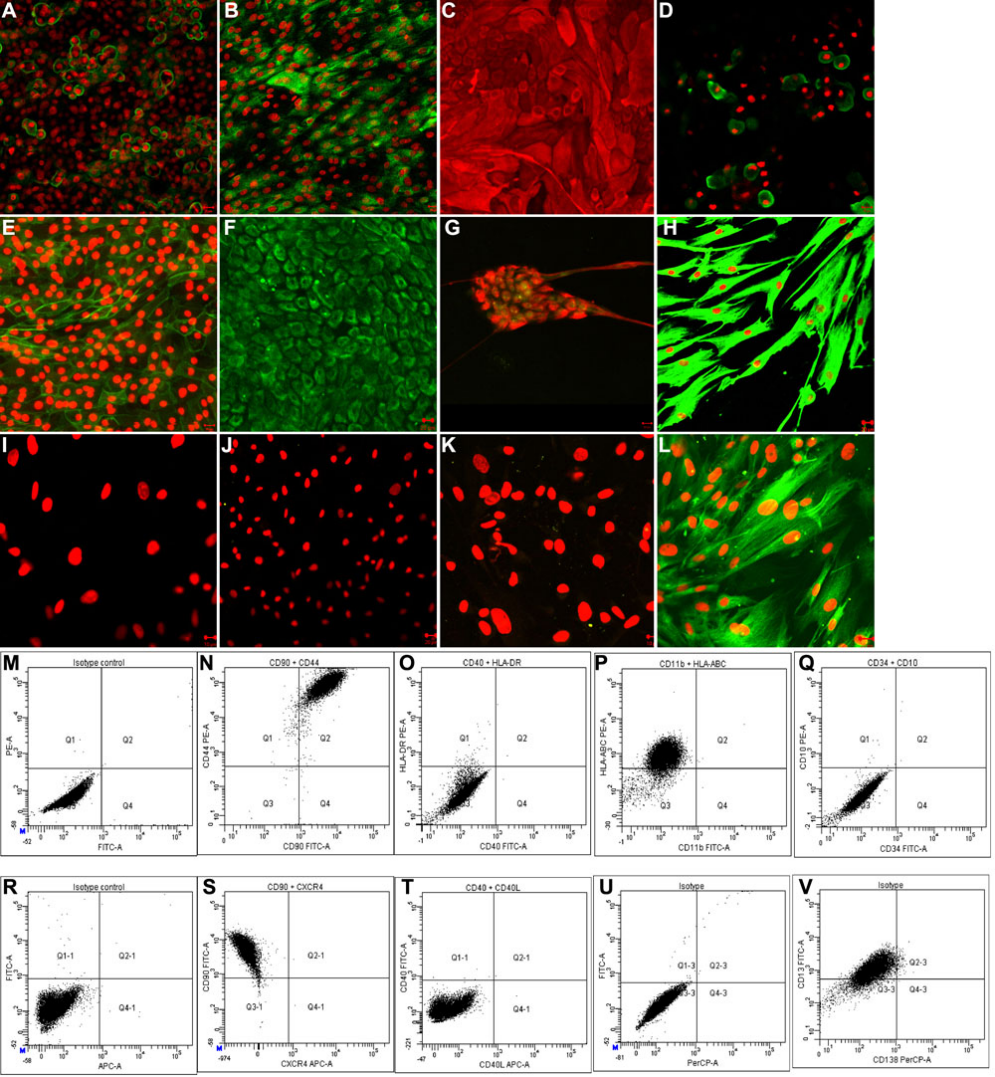
Characterization of limbal explant culture derived epithelial (LEC) and mesenchymal like cells (MC-L). Following immunoctytochemical analysis (**A**-**L**) epithelial cells showing positive for ABCG2 (**A**), CK3/CK12 (**B**; green fluorescence), CK19 (**C**; red fluorescence), CK14 (**D**), E-Cadherin (**E**), vimentin (**F**; green fluroscence), double immunostaining for PAX-6 (green fluorescence) and vimentin (red fluorescence; **G**). Mesenchymal like cells of limbus (**H**-**L**) showing positive for vimentin (**H**) and negative for cytokeratin 3/12 (**I**), cytokeratin 14(**J**), CD34 (**K**), and nestin (**L**). Nuclear staining was performed with propidium iodide (red; **A**, **B**, **D**, **E**, **H**-**L**). Scale bar=20 µm (**A**-**C**, **E**-**H**, and **J**) and 10µm (**D**, **I**, **K**, **L**). Flow cytometry analysis (**M**-**V**) was performed by incubation of the mesenchymal like cells of limbus with the indicated antibodies. **M**: Isotype controls for FITC and PE, **N**: CD90 FITC and CD44 PE, **O**: CD40-FITC and HLA-ABC PE, **P**: CD11b FITC and HLA-ABC PE, **Q**: CD34 FITC and CD10 PE **R**: Isotype control for FITC and APC, **S**: CXCR4 APC and CD90 FITC, **T**: CD40L APC and CD40 FITC, **U**: Isotype control for FITC and PerCP, **V**: CD138 PerCP and CD13 FITC.

The LEC on immunocytochemical analysis, showed immunoreactivity toward ABCG2, CK3/CK12, CK14, PAX-6, CDH1, and vimentin. MC-L were found to be immunoreactive for vimentin and nestin and were negative cytokeratin 3/12 (KRT3/12), cytokeratin 14 (KRT14), and CD45 (Figure 2A-L).

Flowcytometry analysis of MC-L revealed expression of CD90+CD44 (Figure 2N [Q2=98.4%]), CD13 (Figure 2V [Q1–3=86.4%]), and HLA-ABC (Figure 2P [Q1=91.1%]) and were negative or weak expression for CD34 (Figure 2Q [Q4=0.0%]), CD10 (Figure 2Q [Q1=0.1%), CD34+CD10 (Figure 2Q [Q2=0.0%]) CD11b (Figure 2P [Q4=0.0%]), CD40 (Figure 2O [Q4=0.0%]), CD40L (Figure 2T [Q4–1=0.0%]), CD138 (Figure 2V [Q4–3=0.0%]), CXCR4 (Figure 2S [Q4–1=0.0%]) and HLA-DR (Figure 2O [Q1=0.1%]) expression (Figure 2M-V).

### Microarray data analysis

In this study two different samples of LEC, MC-L, and MSC-BM were labeled using (Cy3 and Cy5) dyes and competitive hybridization was performed. The data has been deposited in NCBI’s Gene Expression Omnibus (GEO) with GEO series accession number (GSE16763). The fold change was expressed as average of two different biologic samples. Analysis of the data considering a threefold difference, suggested differential expression of 3,484 genes between LEC and MC-L; 1,579 genes between MC-L, and MSC-BM and 4,837 between LEC and MSC-BM. The differentially/highly expressed genes in LEC, MC-L, and MSC-BM are shown in Table 2. The groups were segregated based on the average fold expression toward one lineage as compared to other, i.e genes that are highly representative of: a) LEC≥25 fold expression as compared to MC-L and MSC-BM; b) MC-L≥15 fold expression as compared to LEC and MSC-BM; c) MSC-BM≥20 fold expression as compared to LEC and MC-L; d) MC-L and MSC-BM≥20 fold expression compared to LEC; e) LEC and MC-L≥10 expression compared to MSC-BM.

**Table 2:** The differential gene expression between LEC, MC-L and MSC-BM, as measured by the fold change difference of the corresponding genes (Ribosomal genes excluded). Abbreviations: LEC – limbal epithelial cells, MC-L – mesenchymal like cells of limbus, MSC-BM – mesenchymal stem cells of bone marrow.

**Genes differentially expressed in LEC (25 fold overexpression in LEC compared MC-L and MSC-BM)**
Keratin 12, T-cell differentiation protein 2 (MAL2), Nebulette, v-myc myelocytomatosis viral related oncogene, ets homologous factor, calbindin 1, Kringle containing transmembrane protein 2, glucosaaminyl (N-acetyl) transferase 2, carcinoembryonic antigen-related cell adhesion molecule 6, Apolipoprotein B mRNA editing enzyme catalytic polypeptide –like 3a, tumor associated calcium signal transducer 1, sciellin, serine peptidase inhibitor kazal type 5, carcinoembryonic antigen-related cell adhesion molecule 1, interleukin 1 alpha, interleukin 18, dual adaptor of phosphotyrosine and 3-phosphoinositides (DAPP1), Transmembrane channel-like 5, claudin 1, defensin beta 1, WAP four-disulfide core domain 5, chemokine (C-X-C motif) ligand 1, dystonin, desmocollin 2, cadherin 1, transforming growth factor alpha, S100 calcium binding protein A8, Serpin peptidase inhibitor clade B member 5, visinin-like 1, interleukin 1 beta, desmoglein 3, matrix metallopeptidase 10, tumor protein p73-like (p63), homeoboxdomain-only protein (HOP), amphiregulin.
**Genes differentially expressed in MC-L (15 fold overexpression in MC-L compared to LEC and MSC-BM)**
Vascular endothelial growth factor receptor 1, cadherin 6, forkhead box F1, glutamate receptor ionotrophic, collectin sub-family member 12, SHC (Src homology 2 domain containing) transforming protein 3 (SHC3), AF052115, BC073929.
**Genes differentially expressed in MSC-BM (20 fold overexpression in MSC-BM compared to LEC and MC-L)**
growth differentiation factor 6 (GDF6), Urea transporter, erythrocyte (SLC14A1), neurotrophic tyrosine kinase, receptor, type 2 (NTRK2), early growth response 2 (Kro × −20 homolog, Drosophila) (EGR2), secreted phosphoprotein 1 (osteopontin, bone sialoprotein I, early T-lymphocyte activation 1) (SPP1), myogenic factor 6 (herculin) (MYF6), collagen, type XI, alpha 1 (COL11A1), olfactomedin 4 (OLFM4), hepatitis A virus cellular receptor 2 (HAVCR2), homeo box A11, antisense (HOXA11S) on chromosome 7, homeobox C9 (HOXC9), HELAD1S mRNA for helicase, phosphodiesterase 1C, calmodulin-dependent 70 kDa, opioid binding protein/cell adhesion molecule-like (OPCML), transcript variant 2, zinc finger, matrin type 4 (ZMAT4),
**Genes differentially expressed in limbal explant culture derived cells (LEC and MC-L) over bone marrow (10 fold difference)**
semaphorin 3D (SEMA3D), matrix metallopeptidase 1 (interstitial collagenase) (MMP1), vitrin (VIT), Lysophosphatidic acid receptor Edg-7 (LPA receptor 3) (LPA-3), keratin 18 (KRT18), transcript variant 1, insulin-like growth factor 2 mRNA binding protein 3 (IGF2BP3), myelin basic protein (MBP), contactin 3 (plasmacytoma associated) (CNTN3),
**Genes differentially expressed in MC-L, MSC-BM over epithelial cells (over 20 fold difference)**
nucleosome assembly protein 1-like 3 (NAP1L3), thymocyte selection-associated high mobility group box (TOX), axin 2 (conductin, axil) (AXIN2), phosphodiesterase 11A (PDE11A), potassium voltage-gated channel, Isk-related family, member 4 (KCNE4), dermatan sulfate epimerase-like (DSEL), chemokine (C-X-C motif) ligand 12 (stromal cell-derived factor 1), G protein-coupled receptor 124 (GPR124), protocadherin 18 (PCDH18), hyaluronan and proteoglycan link protein 1 (HAPLN1), collagen, type V, alpha 2 (COL5A2), alpha-2-macroglobulin (A2M), decorin (DCN), cerebellar degeneration-related protein 1, 34 kDa (CDR1), ependymin related protein 1 (zebrafish) (EPDR1), formin 2 (FMN2), Platelete derived growth factor receptor alpha, frizzled homolog 7, dapper, antagonist of beta-catenin, homolog 3, microfibrillar associated protein 5, lysyl oxidase, integrin, alpha 8, junctional adhesion molecule 2, protein kinase C, alpha, platelet-derived growth factor receptor, beta polypeptide.

Some of the highly expressed genes in LEC include the CD24 (48 and 155 fold overexpression compared to MSC-BM and MC-L, respectively), FOXA1 (27 and 631 fold overexpression compared to MSC-BM and MC-L, respectively) and KRT13 (11 and 15-fold), LAMA3 (7.4 and 7.3), ITGA6 (22 and 10) and CDH3 (6.0 and 7.4). To explore the interdependence of LEC and MC-L, we looked at the growth factor and cytokine profile of these cells (Table 3). LEC showed high expression of growth factors like transforming growth factor alpha (TGF−α), Amphiregulin (AREG), epiregulin (EREG), hepatocyte binding epidermal growth factor (HB-EGF), growth factor receptor-bound protein 14 (GRB14), fibroblast growth factor 11 (FGF11), and cytokines like chemokine (C-X-C motif) ligand 1 (CXCL1), CXCL2. The MC-L showed high expression of growth factors like FGF7, FGF2 and cytokine CXCL12.

**Table 3:** Differential gene expression between LEC and MC-L in growth factors and cytokine related genes.

**Gene**	**LEC versus MC-L**	**LEC versus MSCBM**	**MC-L versus MSCBM**
amphiregulin (schwannoma-derived growth factor) (AREG)	167.6 (Down)	131.25 (down)	3.74 (down)
transforming growth factor, alpha (TGFA)	57.21 (down)	28.49 (down)	
fibroblast growth factor binding protein 1 (FGFBP1)	38.75 (down)	23.011 (down)	
fms-related tyrosine kinase 1 (vascular endothelial growth factor/vascular permeability factor receptor) (FLT1)	31.0 (up)		96.42 (down)
Platelet derived growth factor receptor alpha (PDGFRA)	26.10 (up)	33.93 (up)	
platelet-derived growth factor receptor, beta polypeptide (PDGFRB)	20.52 (up)	21.90 (up)	
insulin-like growth factor binding protein 5 (IGFBP5)	17.65 (up)	14.81 (up)	
growth factor receptor-bound protein 14 (GRB14)	17.23 (down)	5.82 (down)	2.95 (down)
fibroblast growth factor 2 (basic) (FGF2)	13.78 (up)		
fibroblast growth factor 11 (FGF11)	12.83 (down)		
keratinocyte growth factor-like protein 1 (KGFLP1)	10.9 (up)		9.85 (up)
fibroblast growth factor receptor 1 (fms-related tyrosine kinase 2, Pfeiffer syndrome) (FGFR1)	10.50 (up)	8.96 (up)	
insulin-like growth factor 2 (somatomedin A) (IGF2)	9.9 (up)	6.2 (up)	
connective tissue growth factor (CTGF)	9.77 (up)	15.45 (up)	2.3 (up)
pleiotrophin (heparin binding growth factor 8, neurite growth-promoting factor 1) (PTN)	8.54 (up)	9.9 (up)	
insulin-like growth factor 2 mRNA binding protein 1 (IGF2BP1)	8.10 (up)		
fibroblast growth factor 1 (acidic) (FGF1)	8.03 (up)	5.41 (up)	7.41 (down)
Insulin-like growth factor binding protein 4 (IGFBP4)	7.65 (up)	14.2 (up)	2.96 (up)
fibroblast growth factor 7 (keratinocyte growth factor) (FGF7)	7.51 (up)	15 (up)	4.51 (up)
platelet derived growth factor D (PDGFD)	5.97 (up)	12.7 (up)	
fibroblast growth factor binding protein 3 (FGFBP3)	5.68 (up)	4.4 (up)	
insulin-like growth factor binding protein 5 (IGFBP5)	5.61 (up)	5.4 (up)	2.95 (down)
fibroblast growth factor receptor 2 (fgfr2)	4.50 (down)		4.89 (up)
Hepatocyte growth factor precursor (Scatter factor) (SF) (Hepatopoeitin-A)	4.05 (up)	10.06 (up)	
transforming growth factor beta 1 induced transcript 1 (TGFB1I1), transcript variant 2	3.94 (up)	2.97 (up)	
nerve growth factor, beta polypeptide (NGFB)	3.83 (up)		2.6 (down)
chemokine (C-X-C motif) ligand 2 (CXCL2)	79.06 (down)	4.5 (down)	
chemokine (C-X-C motif) ligand 3 (CXCL3)	26.25 (down)	16.0 (down)	3.88 (up)
chemokine (C-X-C motif) ligand 1 (CXCL1)	65.87 (down)	64.9 (down)	
chemokine (C-X-C motif) ligand 11 (CXCL11)	58.344 (down)	14.42 (down)	
chemokine (C-X-C motif) ligand 10 (CXCL10)	7.71 (down)	6.97 (down)	
chemokine (C-X-C motif) ligand 12 (stromal cell-derived factor 1) (CXCL12)	34.26 (up)	82.68 (up)	
chemokine (C-C motif) ligand 26 (CCL26)	11.4 (up)	2.49 (up)	
chemokine (C-C motif) ligand 2 (CCL2)	23.43 (up)		3.48 (down)
chemokine (C-C motif) ligand 13 (CCL13)	12.42 (up)		2.34 (up)
interleukin 1, alpha (IL1A)	78.72 (down)	63.61 (down)	
interleukin 1, beta (IL1B)	48.57 (down)	28.89 (down)	
interleukin 1 receptor, type II (IL1R2)	12.17 (down)	21.03 (down)	3.92 (up)
interleukin 1 receptor antagonist (IL1RN)	20.06 (down)	15.10 (down)	
interleukin 20 receptor, alpha (IL20RA)	210.69 (down)		
interleukin 18 (interferon-gamma-inducing factor) (IL18)	78.26 (down)	56.72 (down)	
interleukin 23, alpha subunit p19 (IL23A)	28.40 (down)	29.29 (down)	
interleukin 11 receptor, alpha (IL11RA)	15.58 (up)		
neurotrophin 5 (NTF5)	14.96 (down)	11.64 (up)	
neurotrophin 3 (NTF3)	16.40 (up)	7.50 (up)	7.48 (down)
nerve growth factor, beta polypeptide (NGFB)	3.8 (up)		
Glial cell line-derived neurotrophic factor precursor (Astrocyte- derived trophic factor 1)	4.24 (up)	6.56 (up)	
GDNF family receptor alpha 1 (GFRA1)	6.5 (up)	6.28 (up)	
brain-derived neurotrophic factor (BDNF)	9.15 (up)	3.69 (up)	8.68 (down)

The analysis of MC-L and MSC-BM showed similar and differential gene expression between the two cells. Various gene ontology terms were picked up and analyzed from the microarray data. The gene ontology terms were classified into groups like osteogenic, chondrogenic, myoblast, adipogenic, MHC-class II related, Homeobox genes, extracellular, and other genes (Table 4).

**Table 4:** Comparisons of genes expressed in MC-L and in MSC-BM cells for selected terms of gene ontology. Abbreviations: LEC – limbal epithelial cells, MC-L – Mesenchymal like cells of limbus, MSC-BM – Mesenchymal stem cells of bone marrow. All the genes are highly expressed (up) except those in brackets (down).

**Gene Name**	**LEC versus MC-L**	**LEC versus MSC-BM**	**MC-L-MSC-BM**
**Osteogenesis**			
Osteonectin	5.8	3.89	
Collagen, type I, alpha 2	10.7	10.15	
Connective tissue growth factor	9.7	15.45	
Collagen, type V alpha 2	16.7	22.2	
Osteopontin		3.8	29.4
Runt related transcription factor 2		5.9	5.5
PDZ and LIM domain 7, transcript variant 4	5.3	3.29	
Gremlin 2	16.1	14.29	
**Myogenesis**			
Transgelin	4.2	5.31	
Meltrin alpha	10	13.78	
Myosin light chain 9, transcript variant 2	4.7	4.87	
Synocoilin 1	8.2	5.7	
Tropomyosin1 (alpha), transcript variant 3	4.7	6.84	
Tropomyosin 2 (beta), transcript variant 2	3.5	3.75	
Caldesmon 1, transcript variant 1	13.8	17.7	
Desmuslin, transcript variant A	26.4	27.9	
Leiomodin 1	8.5	9.29	
**Adipogenesis**			
Leptin Receptor	4.94	60.58	26.74
Leptin		6.24	6.89
Serum amyloid A1, Transcript variant 1	6.8 (down)	4.2 (down)	7.7
CCAAT/enhancer binding protein alpha	4.8 (down)		3.23
**Chondrogenesis**			
Fibromodulin	10.1	13.82	
Decorin, transcript variant A1	27.6	41.45	
Cartilage oligomeric matrix protein		8.4	11.15
Tensin 1	14.9	10.31	
Hyaluronan and proteoglycan link protein	31	47.45	
Collaten, type XI, alpha 1		60.18	46.12
Chitanase 3-like 1		4.72	5.68
**Extracellualr Matrix Components**			
Microfibrillar associated protein 5	24	28.12	
Syndecan 2	12.1	16.7	
Matrix-remodelling associated 5	11.3	7.2	
Chondroitin sulfate proteoglycan 4	8.3	7.5	
Collagen, type VIII, alpha 1, transcript variant 1	8.6	5.39	
**Others**			
Procollagen-lysine 1,2-oxoglutarate 5-dioxygenease 1	3.2	3.3	
Low density lipoprotein-related protein 12	3.43	3.3	
Notch homolog 2	3.78	5.2	
Collagen, type VI alpha 1	3.9	2.39	
Cysteine-rich, angiogenic inducer, 61	4	4.8	
Glial cell line-derived neurotrophic factor precursor	4.2	6.5	
Endoglin	4.29	4.17	
Collagen, type 1, alpha 2	4.3	3.3	
Leukemia inhibitory factor receptor alpha (LIFR)	4.46	5.9	
Neuropilin 1	4.6	4.1	
Colony stimulating factor 1 (Macrophage)	5.6	15.09	
Wingless-type MMTV integration site family, member 5B	5.7	6.06	
Neuronal growth regulator 1	6.13	8.06	
Noggin	6.2	3.7	
Matrix metallopeptidase 2	6.27	6.2	
Collagen, type VI, alpha 3, transcript variant 4	6.27	8.4	
Neuronal PAS domain protein 1	6.38	5.7	
Fibroblast growth factor receptor 1	6.45	8.9	
Neuropilin 1, transcript variant 1	6.7	8.3	
Collagen, type VI, alpha 2	7.4	6.2	
Collagen, type VI, alpha 1	8.0	10.2	
Bone marrow stromal cell antigen	8.2	8.4	
Collagen, type V, alpha 1	8.4	9.3	
Fibronectin 1, transcript variant 7	9.24	10.14	
Fibroblast growth factor receptor 1, transcript variant 1	10.5	7.6	
Collagen, type 1 alpha 2	10.7	10.15	
Angiopoietin 1	14.8	17.1	
Neuronal cadherin	8.4	13.5	

### Validation of microarray using semi quantitative RT–PCR and Real-time PCR

**Figure 3:**
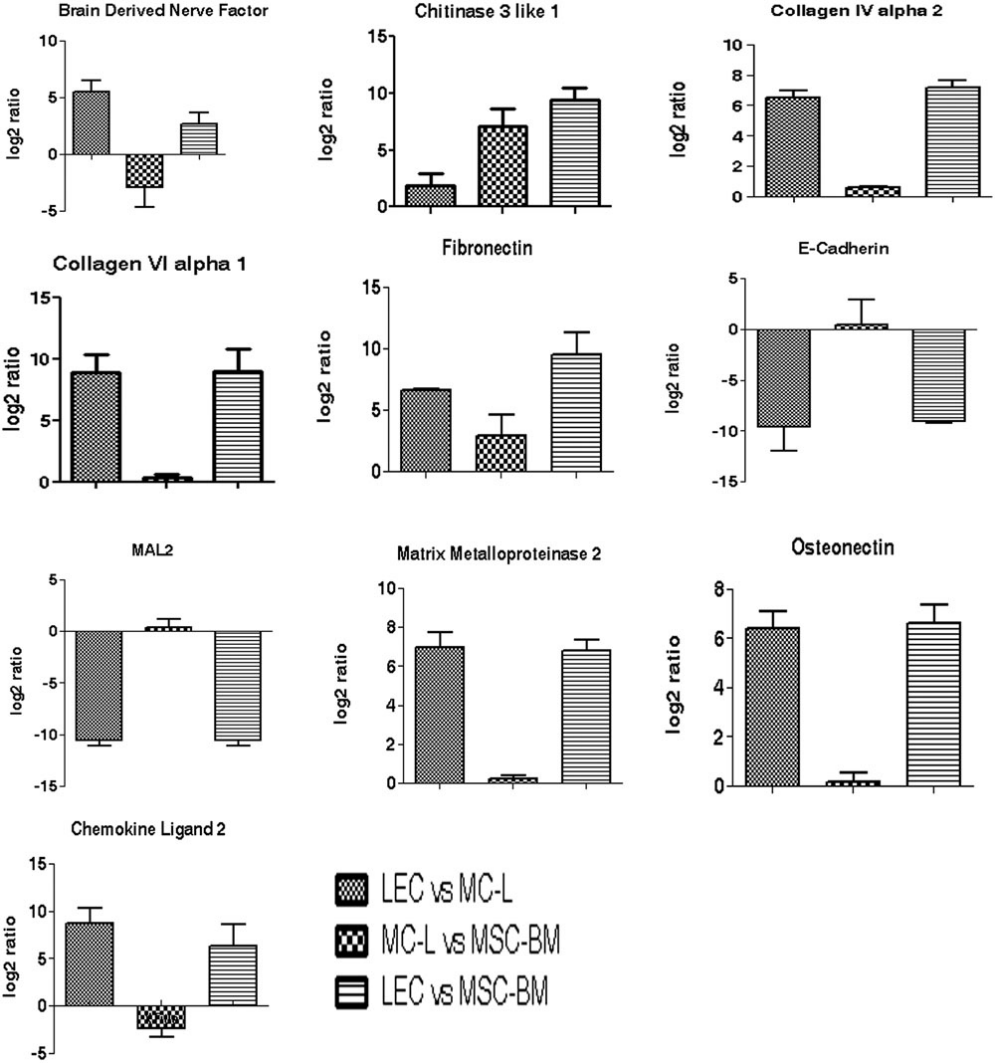
Validation of microarray data by Real time RT–PCR. The individual gene-specific values thus calculated were averaged to mean±standard deviation and fold change was expressed as log 2 ratios (y-axis).

**Figure 4:**
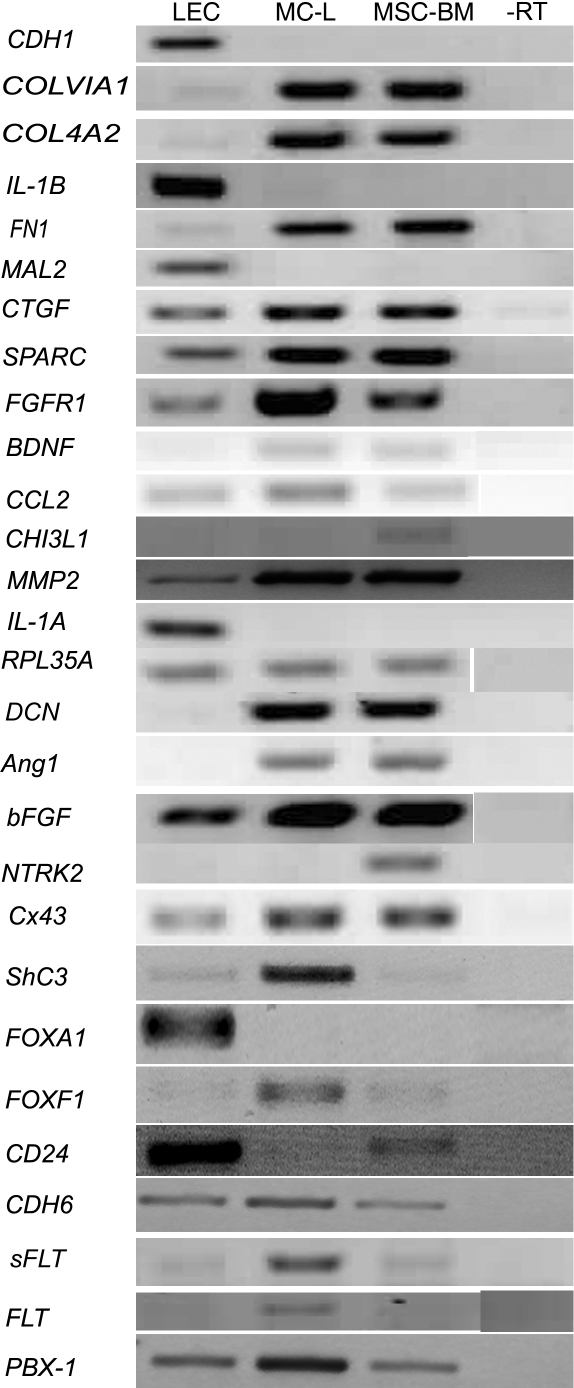
Validation of micro array data by semiquantitative RT–PCR. Reverse transcription polymerase chain reaction analysis of the selected differentially expressed genes. Ribosomal protein large 35 (*RPL35*) was served as an internal control. Abbreviations: LEC – limbal epithelial cells, MC-L – mesenchymal cells of limbus, MSC-BM – mesenchymal stem cells of bone marrow, -RT – no reverse trasncriptase, *CDH1* – cadherin 1 (E-cadherin), *COLVIA1* – collagen 6 alpha 1, *COL4A2* – collagen 4 alpha 2, *IL-1B* – interleukin 1 beta, *FN1* – fibronectin 1, *MAL2* - T-cell differentiated antigen 2, *CTGF* – Connective tissue growth factor, *FGFR1* – fibroblast growth factor receptor 1, *BDNF* – brain derived nerve growth factor, *CCL2* – chemokine ligand 2, *CHI3L1* – chitanase 3 like 1, *MMP2* – matrix metallo peptidase 2, *IL1A* – interleukin 1 alpha, *KRT7*- cytokeratin 7, *DCN* – decorin, *Ang 1* – angiopoientin, *bFGF* – basic fibroblast growth factor, *NTRK2* – neurotrophin tyrosine kinase receptor 2.

To validate the gene expression profiles determined by the microarray analysis, the expression levels of selected genes were analyzed by real time PCR (10 genes; Figure 3) and semi quantitative RT–PCR (28 genes, Figure 4). The gene expression patterns obtained by the two techniques were in good agreement with that from the microarray analysis, indicating high fidelity in microarray data and analytical methods.

## Discussion

Limbal stem cell deficiency has been a challenging clinical problem, the current treatment of which involves replenishing the depleted limbal stem cell pool by either limbal tissue transplantation or use of cultivated limbal epithelial sheets [1-3,24]. As described in our earlier publications [25,26], we established a feeder cell free method of cultivating the limbal explant tissues on denuded human amniotic membrane. Our results show that limbal explant culture derived MC-L when expanded exhibit a spindle shaped, fibroblast-like appearance similar to that of MSC-BM [17]. Though we had no logical explanation for this in the beginning, the revelation of presence of spindle cells prompted us to postulate that these spindle cells in the explant culture system function like “intrinsic feeder cells.” To the best of our knowledge and literature search this is the first study that characterizes the cultured cells (LEC as well as MC-L) from the limbal explant culture, which are directly used for translational research in humans. Isolated MC-L can be distinguished from epithelial cells (lack of expression of KRT3/12, KRT14), fibroblasts (lack of expression of HLA-DR), hematopoietic stem cells (lack of expression of CD34, CD45, CD11b, CD10, CD40, CD40L, and CD138), because they are adherent to the surface of tissue culture flasks and express different cell-surface markers (CD90, CD13, CD105, and CD44).

Genes that show differential expression in the LEC when compared to MC-L and MSC-BM, encode proteins that stabilize epithelial sheets and promote or regulate cell to cell interaction and cell to matrix interaction including keratins (Keratin 13, Keratin 12), laminins (LAMA3, LAMB3), cadherins (CDH3 and CDH1), nebulette, epiregulin, calbindin 1 28 kDa, desmosomal components (DSG3, DSC2), matrix metallo peptidase 10, Serine peptidase inhibitor clade B5, and carcinoembryonic antigen-related cell adhesion molecule 1. In addition, LEC showed high expression of known basal markers (TP73L [p63], ITGA6, epiregulin, and HOP) and differentiated epithelial markers (CDH1, KRT12) [27]. Immunocytochemical analysis showed the expression of limbal epithelial stem cells markers ABCG2, vimentin, KRT14, and KRT19 and also expressed the differentiated epithelial markers CDH1 and KRT3/12 on cultivated LEC. This further supports the fact that cultivated LEC cells on dhAM in a feeder cell- free culture technique, contain a distinct population of stem cells and differentiated cells which serve to replenish the depleted limbal stem cells when transplanted to the diseased eye [1,2,25]. Some of the high expressed transcripts in the limbal epithelial cell cultures include CD24, a surface molecule that has been used to identify different types of human stem cells [28]; OTX1, a transcription factor is expressed in the presumptive ciliary body and iris and has been shown to be essential for development of these tissues [29], and FOXA1, an endodermal stem cell marker [30]. While it could be critically argued that the study does not represents the specific signature of the limbal stem cells alone, this data are valuable in contribution to data on entire population of cultured limbal epithelial cells that are used for clinical transplantation. This further supports strong evidence that the cultivated LEC cells contain a distinct population of stem cells and differentiated cells which serve to replenish the depleted limbal stem cells when transplanted to the diseased eye – it represents the signature of cultured limbal cells inclusive and not exclusive of stem cells.

Cytokine and growth factor signaling is an important determinant of the functional state of these cells and therefore we evaluated this relationship between LEC and MC-L [15]. The most important growth factors for normal human keratinocyte proliferation are member of EGF family, including TGFA, HB-EGF, ER, and AR and these act in an autocrine manner [31]. Our data also reveals the high expression of these four EGF members in LEC. The fibroblast growth factors, FGF1 and FGF2, are well characterized growth factors known for their mitogenic effect on several cells derived from neuroectodermal or mesodermal origins. FGF1 and FGF2 (mitogenic to corneal and limbal epithelium) [32], keratinocyte growth factor (FGF7), epithelium specific growth factor (mitogen for several epithelial cells including limbal epithelial cells) [15] were highly expressed in MC-L. Interestingly, and as expected, their corresponding receptors FGFR1 were expressed in MC-L and FGFR2 in LEC. The proinflammatory forms of IL-1 (IL1A and IL1B) expression in LEC play significant roles in ocular surface immune and inflammatory responses and wound healing [33]. The highly expressed chemokine observed in LEC include CXCL1, 2, 3, 10, and 11 and in MC-L include CXCL12, CCL26, CCL2, and CCL13. The intense expression of chemokine ligand CXCL12 (Stromal cell derived factor 1) in MC-L is similar to the study by Tristan and coworkers [34]. This factor might exert physiologic effects on the cornea and could be involved in pathological conditions such as corneal angiogenesis [34]. The neurotrophic factors have been reported to play important roles in maintaining stem cells in the limbus [35]. We also noted a high expression of neurotrophic factors like neurotrophin 3, nerve growth factor and brain derived growth factor in MC-L while neurotrophin 5 was highly expressed in LEC. Glial derived neurotrophic factor (GDNF) and its receptor GDNF receptor alpha 1 were highly expressed in the MC-L and MSC-BM similar to the observations made by Qi and coworkers in limbal cells [35]. All these features support our hypothesis that the limbal epithelial cells and stromal cells play a complementary role not only in vivo but also in vitro in the explants culture system.

Genes highly expressed (Table 2) in MC-L include fetal kidney cadherin (CDH6), vascular endothelial growth factor receptor 1 (VEGFR1 or FLT1) glutamate receptor ionotrophic (GRIA3), collectin subfamily member 12, transcription factor forkhead box F1 (Foxf1), src homology 2 domain contataining transforming 3 (SHC3), oxytocin receptor, and unknown genes AFO52115 and BCO73929. These genes with such higher expression (>15 fold) can be considered as the markers of mesenchymal like cells of limbus. The FOXF1 is a transcription factor which is expressed in mesenchymal cells of the lung, liver and gall bladder and is shown to be involved in mesenchymal cell migration without changes in cell proliferation and cells survival [36].

An interesting observation was the high expression of receptors neurophilin 1, platelet derived growth factor receptor alpha, and leprecan-like 2 in MC-L, which is similar to MSC-BM [37]. This study supports the characteristics of mesenchymal cells (MSC-BM, and MC-L) that were previously identified in MSCs, such as vimentin, fibronectin, collagen Type I and III, collagen type VI, light chain of myosin 9, and matrix metallopeptidase 2 [37-40]. The genes which show similar gene expression in MC-L and MSC-BM are those which are related to extracellular components, cell adhesion molecules (microfibrillar associated protein 5, syndecan 2, matrix-remodelling associated 5, chondroitin sulfate proteoglycan 4, and collagen 8 alpha 1) and the genes related to osteoblasts (osteonectin, collagen type 1, connective tissue growth factor, and OB-cadherin), chondrocytes (fibromodulin, decorin, tensin 1, hyaluronan, and proteoglycan link protein) and myoblasts (transgelin, sarcoglycan epsilon, caldesmon 1, leimodin, and meltrin alpha; Table 4). The MC-L also expressed the products characteristics of hematopoiesis-supporting stroma, including fibulin-1, fibulin 2, collagen type VI, and stromal cell-derived factor, in the same level as MSC-BM thus supporting our hypothesis that these cells possibly act as intrinsic feeder cells or nurture cells in explant culture system. Nevertheless, some differences were observed between expression profiles of MC-L and MSC-BM. Among the genes that were exclusively or expressed at higher levels by MSC-BM are growth differentiation factor 6, neurotrophic tyrosine kinase receptor 2, urea transporter erythrocyte, and myogenic factor 6. Other genes highly expressed at higher levels in MSC-BM include chondrogenesis related genes (cartilage oligomeric matrix protein, collagen 11 alpha 1, and chitinase 3 like 1), osteogenic related genes (runt related transcription factor 2 and osteopontin) and adipogenic related genes (CCAAT/enhancer binding protein alpha, leptin, and Serum amyloid A1 transcript variant 1). The MSC-BM are more committed to the osteoblastic, chondrogenic and adipocytic lineages. This suggests that in addition to some common signatures of niche supporting cells, mesenchymal cells from different sources possibly carry tissue specific signatures, which reflect their tissue of origin. The limitations of study would be the sample size used for microarray (n=2) and potential contamination of stromal cells in limbal epithelial cells cultures.

In summary, this study highlights the gene expression profile of cultivated limbal epithelial cells, mesenchymal cells from limbal stroma obtained from an ex-vivo expanded, feeder cell-free, limbal explant tissue culture system. Their lineage specific signatures, evidence of interdependent pathways with limbal epithelial cells, striking resemblance to the signature of bone marrow derived mesenchymal cells support our hypothesis that the limbal stromal cells act like intrinsic feeder cells or the nurture cells, similar to bone marrow derived mesenchymal cells and could possibly be an important component of limbal niche.
